# Understanding the Neuroscience Underpinnings of Obesity and Depression: Implications for Policy Development and Public Health Practice

**DOI:** 10.3389/fpubh.2021.714236

**Published:** 2021-08-20

**Authors:** Brenda Robles, Tony Kuo, Adriana Galván

**Affiliations:** ^1^Department of Community Health Sciences, Fielding School of Public Health, University of California, Los Angeles, Los Angeles, CA, United States; ^2^Department of Epidemiology, Fielding School of Public Health, University of California, Los Angeles, Los Angeles, CA, United States; ^3^Department of Family Medicine, David Geffen School of Medicine at the University of California, Los Angeles, Los Angeles, CA, United States; ^4^Population Health Program, Clinical and Translational Science Institute, University of California, Los Angeles, Los Angeles, CA, United States; ^5^Department of Psychology, Brain Research Institute, University of California, Los Angeles, Los Angeles, CA, United States

**Keywords:** neuroscience, obesity, depression, public health interventions, policies, systems, environments, program planning

## Introduction

Obesity and depression are widespread problems that have major public health implications, both in the United States and abroad. An assessment of the World Health Organization's 2015 Global Health Estimates found that obesity-related chronic conditions (e.g., heart disease, stroke, diabetes) were among the top ten leading causes of death globally (1). Depression, which is associated with heightened mortality risk (2), is the leading cause of disability worldwide (3). The burden of these two conditions has grown in recent years. Between 1990 and 2017, the global incidence of depression increased by nearly 50% (4). The global prevalence of excess body weight has also continued to rise among adults in most countries (5).

Excess adiposity and depressive symptomatology, if unabated, can each or in combination result in deleterious consequences to individuals and their communities. Obesity, for example, is associated with an array of chronic medical conditions, including cancer, hypertension, type 2 diabetes, coronary heart disease, and other forms of cardiovascular disease (6). Direct medical costs associated with obesity (and with overweight status) have been estimated to account for more than 20% of all health care spending in the United States (U.S.) (7). Similarly, the social and economic ramifications of depression also appear to be substantial around the world (3, 8).

In clinical practice, there is growing recognition that when an individual is diagnosed with obesity or depression, healthcare practitioners should check for the presence of and/or treat the other condition (9). This call to action is bolstered by established evidence that these two conditions co-occur (9). Several studies, for instance, have found that adults who are overweight/obese are at increased risk of depression (10). Yet, public health practice often falls short in addressing them. This represents a missed opportunity to intervene.

In this article, we discuss the need for public health to expand its scope and understanding of neuroscience, learning from this discipline to include depression detection and management as part of a more holistic approach to preventing obesity. Namely, we argue that breaking existing silos between the fields of public health and neuroscience may help strengthen the effectiveness of policy, systems, and environmental change interventions (PSEs) which are frequently used to combat obesity at the population-level. To facilitate this dialogue, we first reviewed the current literature on the neural correlates of obesity and depression. We then highlight the potential advantages of considering these correlates in developing PSEs and other interventions for obesity prevention.

## What is Known in the Literature

### Neural Correlates of Obesity

Neuroscience is a field that elucidates the underlying mechanisms that motivate individuals to eat, which in excess, may result in overweight and obesity. In essence, the brain plays a central role in controlling hunger and regulating eating behaviors (11). The prefrontal cortex or “control” region of the brain, in addition to other functions, helps individuals control their behavior, inhibit their impulsive responses, and evaluate and make decisions about environmental stimuli (12). Several studies have found that overeating (i.e., a behavior linked to overweight/obesity) is attributed to impaired inhibitory control in networks of the brain where the prefrontal cortex is a key node (13).

The limbic system, a set of brain structures connected to the prefrontal cortex, also shapes individuals' motivation behaviors (14). For example, there is evidence that the mesolimbic structures of the brain, and the mesocorticolimbic circuitry or “reward pathway” of the brain, are responsible for the hedonic aspects of eating and incentive salience in food motivation behaviors (15). Other core brain regions associated with dietary self-control include the anterior insula, middle frontal gyrus, supplementary motor cortex, parietal cortices, and fronto-stratrial region (16). Moreover, neurotransmitters (e.g., the hormones leptin and ghrelin) are implicated in the gut-brain reward axis which affects neural functions and controls individuals' eating behaviors and thus obesity status (17).

A growing body of research highlights the intersections between brain activation, food motivation, and eating behaviors (i.e., those characterized by self-control and the ability to delay gratification). For example, adult binge eaters appear to have a lower activation in the fronto-striatal (limbic) region of the brain, and greater trait impulsivity and lower inhibitory control abilities, compared to non-binge eaters (18). It also appears that adolescents with food addiction experience this condition due to hypo-activation in areas of the brain that inhibit control (19). Overall, such studies implicate the brain in food motivation behaviors that can put individuals at risk for excess adiposity.

Neuroscience research also explains the role that stress can play in eating behaviors and on obesity-related outcomes. Stress is considered a common risk factor for both obesity and addiction (20), and there is strong empirical evidence that stress may lead individuals to engage in dietary behaviors that put them at risk for this condition (21). Stress may activate certain brain regions, which could explain conditions such as stress-induced overeating and obesity in adult patients with coronary artery disease (22). Mood may also interact with stress and, in turn, influence what people eat (20). The complex and synergistic relationships between physiological, environmental, and cognitive factors that influence food consumption behaviors of adults and children eating are highlighted by recent advances in neuroscience research (23).

### Neural Correlates of Depression

The etiology of depression is complex, as evidenced by past and recent studies. For example, gene-environment interactions have been linked to depression (24). Meanwhile, others have argued that this mental health condition occurs as a result of alterations in spine synapse connectivity in certain areas of the brain (25) or due to structural and/or functional brain abnormalities (26). There is also growing recognition that depression is a neural circuit-based disease (27) and that different areas of the brain contribute to the development of this condition (28). For example, individuals with major depression may have abnormally reduced activity in the medial and lateral prefrontal cortex (i.e., brain regions that regulate emotions) (29). Other studies have found that individuals with mood disorders such as depression exhibit differences in emotional regulation than individuals with anxiety disorder, highlighting distinctions in neural recruitment of emotional or regulatory brain (30).

Several studies have also used functional magnetic resonance imaging (fMRI) or functional neuroimaging to better understand depression in various adult populations (31–35). For example, a study employing fMRI found that individuals with major depressive disorder exhibit greater activation of the amygdala insula and ventrolateral prefrontal cortex when exposed to social exclusion than those without depression (33). Previous neuroimaging studies have found that cognitive-behavioral therapy, a popular psychotherapy treatment, may decrease “resting state activity in the dorsal ACC” (34), and suggest that such a therapy may help with emotion regulation (35). Neuroimaging studies are not limited to adults and neuroimaging techniques have also been used to examine depression among youth (36, 37).

### Neural Linkages Between Obesity and Depression

Neuroscience research has increasingly become a resource for scientists and health professionals to examine the relationships between obesity and depression. There is emerging data which suggest that neural adaptations in brain circuitry may explain the associations between obesity and depression/depressive symptoms (38, 39). For example, both of these conditions may occur due to the loss of gray matter in the same medial prefrontal cortex of the brain (40). Abnormal inflammation in the brain has also been implicated in both obesity and depression pathophysiology (41). Moreover, data suggests that depression may be rooted in adiposity-related inflammation within the brain (42) and that the gut-brain axis plays a key role in the development of depression (43, 44) and obesity (45).

## Recent Obesity Prevention Efforts at the National and Local Level in the United States

Although correlates such as neural pathways to food motivation can offer important insights for policy development and intervention design, most obesity prevention efforts in the United States do not apply neuroscience in their implementation, choosing to focus primarily on mitigating structural or environmental barriers to healthy eating (46). Unfortunately, data on these structural-level interventions have been mixed, suggesting this socio-ecological approach may be insufficient for fully changing individual consumption behaviors. Various systematic reviews have found an absence of clear evidence demonstrating the effectiveness of these interventions (primarily PSEs) in significantly improving population-level obesity or related health outcomes (47, 48). It is possible that increasing the availability of “healthier” foods does not guarantee that individuals will select them, especially if these individuals are used to consuming inexpensive and highly palatable foods. Sugary and nutrient-poor beverages such as soda, for instance, put individuals at risk for obesity (49). There is accumulating evidence that consuming sugary beverages engender a physiological response similar to that of drugs (50). In contrast, healthy foods that reduce individuals' obesity risk (e.g., fruits and vegetables) do not appear to produce a similar response, but they do generally cost more to purchase relative to the unhealthier food options, creating an economic disincentive (51).

To summarize, recent obesity prevention interventions may be limited in their reach and effectiveness because they fail to consider the underlying neuropsychological mechanisms that may prevent individuals from eating healthy. After all, many have argued that tailoring obesity prevention interventions to specific groups require public health to understand the drivers of food consumption behaviors (52). In this regard, federal and local obesity prevention efforts have fallen short in optimizing these drivers of food decisions. Los Angeles County serves as an example of a metropolitan region that has not traditionally sought to intervene in mental health (including depressive symptomatology and related neurobiological factors) and incorporate this aspect of care into ongoing prevention efforts to reduce overweight/obesity (see Table 1).

**Table 1:** Obesity prevention initiatives implemented in Los Angeles County, 2010–2016.

**Initiative**	**Duration of Funding**	**Federal funding amount**	**Policy, systems and environmental change interventions**	**Neural and Psychological Drivers of Lifestyle Behaviors**
Communities Putting Prevention to Work	2010–2012	$16 million	• Adoption and implementation of: ° Healthy food and beverage standards in worksites and schools ° Nutrition and physical activity guidelines for preschools ° Institutional policies to support breastfeeding • Implementation of training to increase school capacity and teacher skills to teach quality physical education • Adoption and strengthening of joint-use agreements in school districts • Adoption of land use practices to increase pedestrian activity and biking	Not required in the project work plan.
Community Transformation Grants	2011–2015	~$30 million	• Adoption and implementation of patient-centered medical home (team-based care approach) to deliver high-impact clinical prevention services • Establishment of practice-based network to promote sharing of lessons learned and dissemination of best practices on clinical preventive services in County of Los Angeles health and public health centers. • Expansion of supplemental nutrition assistance program benefits at farmers' markets serving low-income communities • Establishment of collaborations with school districts in low-income communities to increase freshly prepared school meals and to increase student participation in these meal programs • Development of a health and wellness element included in the General Plans of the City of Los Angeles and other municipalities/communities • Expansion of the Los Angeles County Parks After Dark Program	Although emotional health was a focus area which could be selected, it was optional and not a primary focus of the initiative or the project work plan.
First 5 LA Early Childhood Obesity Prevention Initiative	2012–2016	~$41 million	• Dissemination of the following to families with children ages 0–5: ° Nutrition and physical activity education and resources ° Parent nutrition education and skills-building • Implementation of a County-wide media and targeted social marketing campaign aimed at families and caregivers of children ages 0–5 • Implementation of menu changes that expanded healthy children's meal menu options or reduced the portion size of the meals • Implementation of obesity prevention protocols for children ages 0–5 that included routine body mass index measurement and tracking, nutrition and physical activity education, and more intensive case management of overweight, obesity, or other factors	Not required in the project work plan.
Nutrition Education and Obesity Prevention Program	2012–2016	~$42 million	• Implementation of school wellness policies • Implementation of healthy retail • Promotion of edible gardens • Adoption and implementation of worksite wellness policies • Adoption and implementation of healthy food and beverage standards	Not required in the project work plan.

## Discussion

So where do we go from here? Now, more than ever, there is a need for public health to find effective solutions to combat obesity and related chronic conditions. This is especially relevant to population health given the 2019 outbreak of the novel coronavirus disease (COVID-19), which has exposed the deleterious nexus the brain, psychological distress, and chronic conditions like obesity. In the COVID-19 era, individuals with hypertension and/or diabetes, conditions associated with obesity (53, 54), appear to be at increased risk for more severe COVID-19 (55). Individuals with these conditions are generally more likely to be hospitalized and to experience greater medical complications from this infection (56). It is also important to consider the pandemic's impact on mental health, especially depression, which appears to be relatively pronounced; this is in part due to the social isolation caused by the lockdowns and mandates to practice social distancing (57). Collectively, the COVID-19 situation offers a cautionary tale about the inter-connectedness of infectious, psychological distress, and chronic conditions, lending further support for public health to expand its reach and scope to encourage cross-pollination with disciplines such as neuroscience, to better serve the population and improve overall population health in the U.S. and across the globe.

## Author Contributions

BR conceptualized and drafted the initial article. TK provided subject matter expertise and guidance on chronic disease interventions described in the article. AG provided subject matter expertise related to the neuroscience literature discussed in this article. All authors helped to draft or revise the article.

## Conflict of Interest

The authors declare that the research was conducted in the absence of any commercial or financial relationships that could be construed as a potential conflict of interest.

## Publisher's Note

All claims expressed in this article are solely those of the authors and do not necessarily represent those of their affiliated organizations, or those of the publisher, the editors and the reviewers. Any product that may be evaluated in this article, or claim that may be made by its manufacturer, is not guaranteed or endorsed by the publisher.
